# Characteristics of paediatric foot arches according to body mass among primary school students in Wrocław, Poland

**DOI:** 10.1186/s12887-022-03699-z

**Published:** 2022-11-10

**Authors:** Sara Górna, Katarzyna Pazdro-Zastawny, Alicja Basiak-Rasała, Mateusz Kolator, Joanna Krajewska, Tomasz Zatoński

**Affiliations:** 1 Department of Physiology and Biochemistry, Poznan University of Physical Education, 61-871 Poznań, Poland; 2 Department of Otolaryngology, Head and Neck Surgery, Wroclaw Medical University, 50-367 Wrocław, Poland; 3 Department of Social Medicine, Wroclaw Medical University, 50-367 Wrocław, Poland; 4 Biegaj Dla Zdrowia Foundation, Wrocław, Poland

**Keywords:** Children, Paediatric foot, Podoscan 2D

## Abstract

**Background:**

The purpose of this study, conducted within the framework of the project entitled ‘Let’s get the kids moving’, was to determine the associations between children’s longitudinal and transverse foot arch and their weight, gender and age.

**Method:**

The pro-health initiative ‘Let’s get the kids moving’ was created by researchers of the Medical University and the Run for Health Foundation. A total of 655 children (51.5% boys) aged 7 to 10 years from primary schools in south-western Poland participated in the study. The mean age of the subjects was 8.7 ± 0.8 years. In all the children, we assessed anthropometric measurements (weight and height) and the longitudinal and transverse arch of the foot under the load of their weight. A two-dimensional foot scanner (Sensor Medica, Italy) was used to examine the plantar part of the children’s feet.

**Results:**

The data collected from the 655 subjects revealed that excessive weight predisposed them to less longitudinal and transverse arching. The foot shape was not differentiated by gender or age.

**Conclusions:**

Screening school-aged children’s footprints can detect abnormalities in the shape of children’s feet early on, which allows for early diagnosis of functional or structural flatfoot in children.

## Background

The human foot is a complex static/dynamic structure that holds the body’s weight [1]. The foot’s core system consists of three subsystems – neural, active and passive – which provide the stability and flexibility that are indispensable for everyday life. Proper functioning of the foot depends on appropriate shape and tension of two transverse and five longitudinal arching and morphological structures [1]. An efficient core system is essential for the proper functioning of the foot, allow it to adapt to changing structure and load conditions [2].

As a child grows and matures, the parameters of their feet change [3] and their neuromuscular control matures [4]. Arch-forming structures are already beginning to develop in infants, when they spontaneously grab their feet. Until the age of two, a child’s foot on the medial side from around the metatarsophalangeal toe to the talocalcaneonavicularis joint is filled with Spitzy’s fat pad [2]. This fatty pad reduces the pressure per unit area of the feet. Starting at around two years of age, the medial foot arch begins to be noticeable. The fatty pad disappears by about the age of five, and flat and/or valgus feet almost completely disappear by the age of seven [5]. Girls reach medial longitudinal arch (MLA) stability earlier than boys [6].

The prevalence of overweight in the among European children remains high [7]. Excessive body mass index (BMI) may contribute to disproportional pressure on the midfoot area and MLA in a standing position [3]. The structures of the musculoskeletal system in developmental age are unable to compensate for this excessive load [3]. Foot dysfunction resulting from various musculoskeletal disorders can prevent physical activity in children and can thereby promote obesity [8]. Excessive BMI changes the foot loading when walking or running, and might lead to musculoskeletal deformity [9]. Slight musculoskeletal dysfunction may contribute to changes in the lower extremity biokinematic chain. This could also apply to the foot’s motility and disturbances to body balance in a standing position [10].

López-López et al. revealed that foot problems have a negative impact on quality of life (QoL) [11]. In another study, specific foot and general health-related QoL did not seem to be influenced by the foot arch height [12]. There is a need for programmes that promote foot health, as the study revealed that arch height has a negative impact on QoL [13].

The purpose of this cohort study was to compare the longitudinal and transverse arching of the feet, of pupils aged 7 to 10 years from primary schools in Wrocław, Poland with their weight, age and gender. The continuation of this study which has been conducted since 2019 is the Population CohorT StUdy of Wroclaw CitizEns (PICTURE) project. This is a comprehensive survey study of children and their parents concerning their health status, the environmental and social factors that influence lifestyle and the occurrence of risk factors.

## Methods

### Study design

This was an observational cohort study designed to assess the correlation between the longitudinal and transverse arching of the feet of Polish pupils, and their weight, age and gender. To evaluate the association precisely, the anthropometric measurements of each child were carried out individually by trained physiotherapists using the same type of measuring devices in all participants, as described below.

The study was conducted within the framework of the project entitled ‘Let’s get the kids moving’, which promotes a healthy lifestyle and physical activity and focusses on the problem of overweight and the resulting diseases in children. The initiative – created by researchers of the Medical University and the Run for Health Foundation – aims for parents, children and teachers to promote a healthy lifestyle in children. Ethical approval for the study was obtained from Wrocław Medical University’s Bioethics Committee (approval no. KB-738/2018). The study was conducted following the Declaration of Helsinki and the recommendations of Strengthening Reporting of Observational Studies in Epidemiology (STROBE).

### Participants

**Fig. 1:**
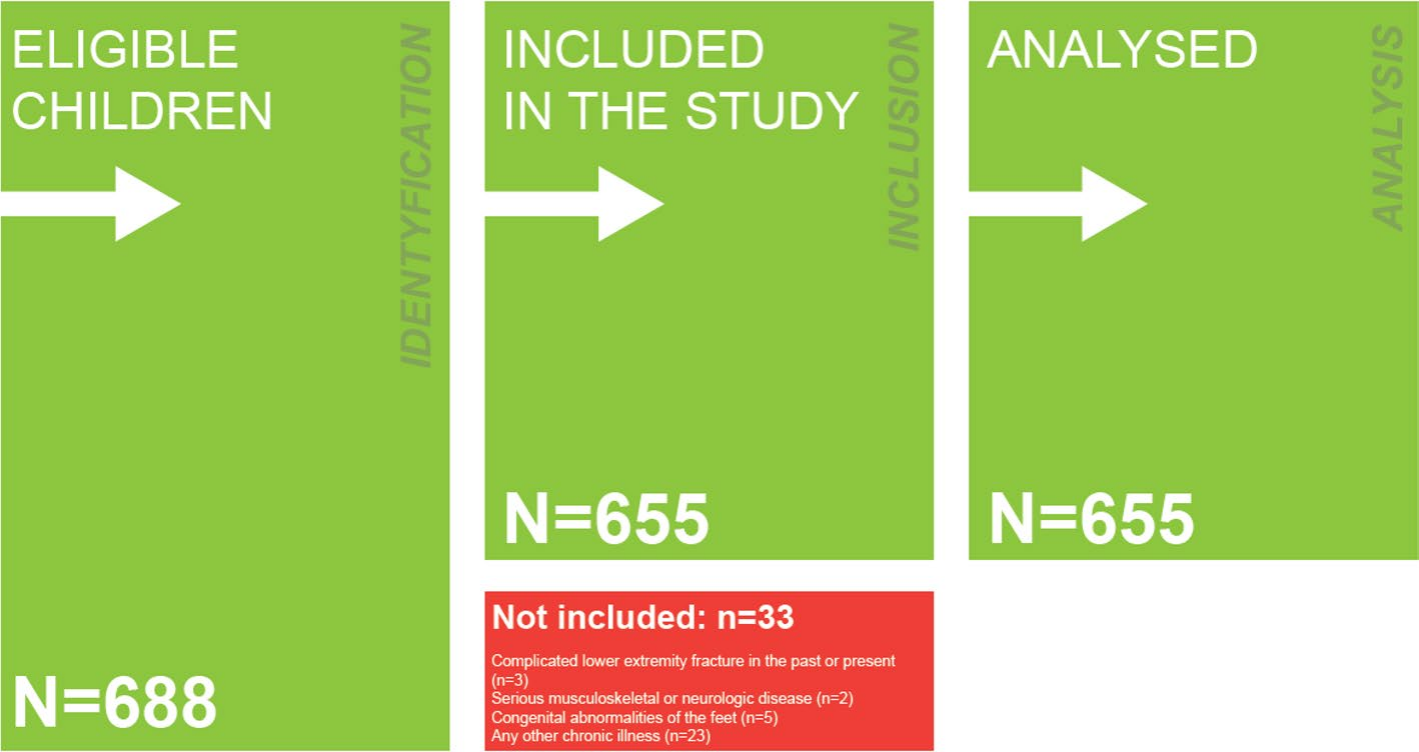
Flow chart of the selection of participants included in the study

The caregivers of children from five public primary schools in Wrocław received written information about the study before the examination started. It was briefly explained that participation in the study was voluntary. The inclusion criteria for the study were written, informed parental consent; an age of between 7 and 10 years; and primary school attendance. Written consent was obtained from 688 parents. The exclusion criteria of the study were complicated lower extremity fractures (past or present); serious musculoskeletal or neurologic diseases; congenital abnormalities of the feet; or any other chronic illness. The study group consisted of 655 children after 33 children (4.80%) were excluded. The study characteristics are shown in the flowchart in Fig. 1.

In our study, we used a probability sampling technique – stratified random sampling –to focus on public primary schools in Wrocław (an urban area in south-western Poland). Five of the city’s 53 public schools were selected using a computer-generated programme that produces lists of numbers at random.

### Measures

#### Weight category

The children’s weight and height were measured using an identical medical weight scale with a stadiometer in all five schools. The anthropometric measurements (weight and height) were taken by physiotherapists in a place designated by the schools’ administrators (a gymnasium or classroom). The anthropometric measurements of each child were carried out individually. Data collection took place between March and June 2018 during school days. Measurements were taken in the mornings between 8.00 am and 1.30 pm. During the examination each child was dressed in light clothing and socks (without shoes). Weight was measured with electronic medical scales (Charder MS 6110, Taiwan) with an accuracy of 0.1 kg. The scales meet directive MDD 93/42 EEC for medical devices. The children’s height was measured using a wall-mounted stadiometer (HM-202P, Taiwan), and the results were accurate to 0.1 cm.

Based on the weight and height measurements of the children, the nutritional status index (Cole’s Index [CI]) was calculated. Cole’s index adjusts the BMI distribution for skewness and allows BMI in individual subjects to be expressed as an exact centile or SD score [14].

The actual body weight and height were compared with the body weight and height read from centile grids (corresponding to the 50th centile for the age and sex of the child). The CI levels were as follows: underweight, normal body weight, overweight and obese. Based on the criteria of the index, the following values were adopted: 90%-–109% was: normal values, 110%-–119%: was overweight, 120% and above: and above was overweight and, range below 90% was underweight in the range below 90% were adopted [15, 16].

### Foot anthropometrics

The plantar part of the foot was examined using a two-dimensional (2D) FootCAD PodoScan (FreeMed, SensorMedica, Italy). All data were recorded with a compatible software programme, FreeSTEP BASIC (version 1.4.01), which allows for manual or automatic measurements. The FootCAD PodoScan dimensions were 623 × 400 × 133 mm, with a maximum scanning area of 304.8 mm × 431.8 mm (W × L). The scanning speed of each footprint was 8.5 s. The device is fitted with a charge coupled device with a cold cathode, which guarantees a quality footprint (optical resolution: 1600 dpi) [17].

During the examination, the each child stood on the PodoScan surface with both bare feet with fully weight-bearing their weight, in an upright position looking straight ahead. The next step after scanning the feet was the to manually evaluation of the medial longitudinal and transverse arches in FreeSTEP BASIC software. To identify the MLA were used the following indicators were used:

- Clarke’s angle (CL angle) – the angle between a tangent to the medial edge of the foot (the most extreme point on the head of the first metatarsal bone [point mtt] and at the medial edge of the heel [point A]) and a line connecting the deeper forefoot part of the footprint (point B) with point mtt, expressed in degrees [^o^]
- Sztriter–Godunov index (K-y index) – determines the ratio of the length of the shaded part of the footprint (line CD) to the length of the shaded and unshaded parts of the footprint (line BD), measured at the height of the centre of the MLA

**Fig. 2:**
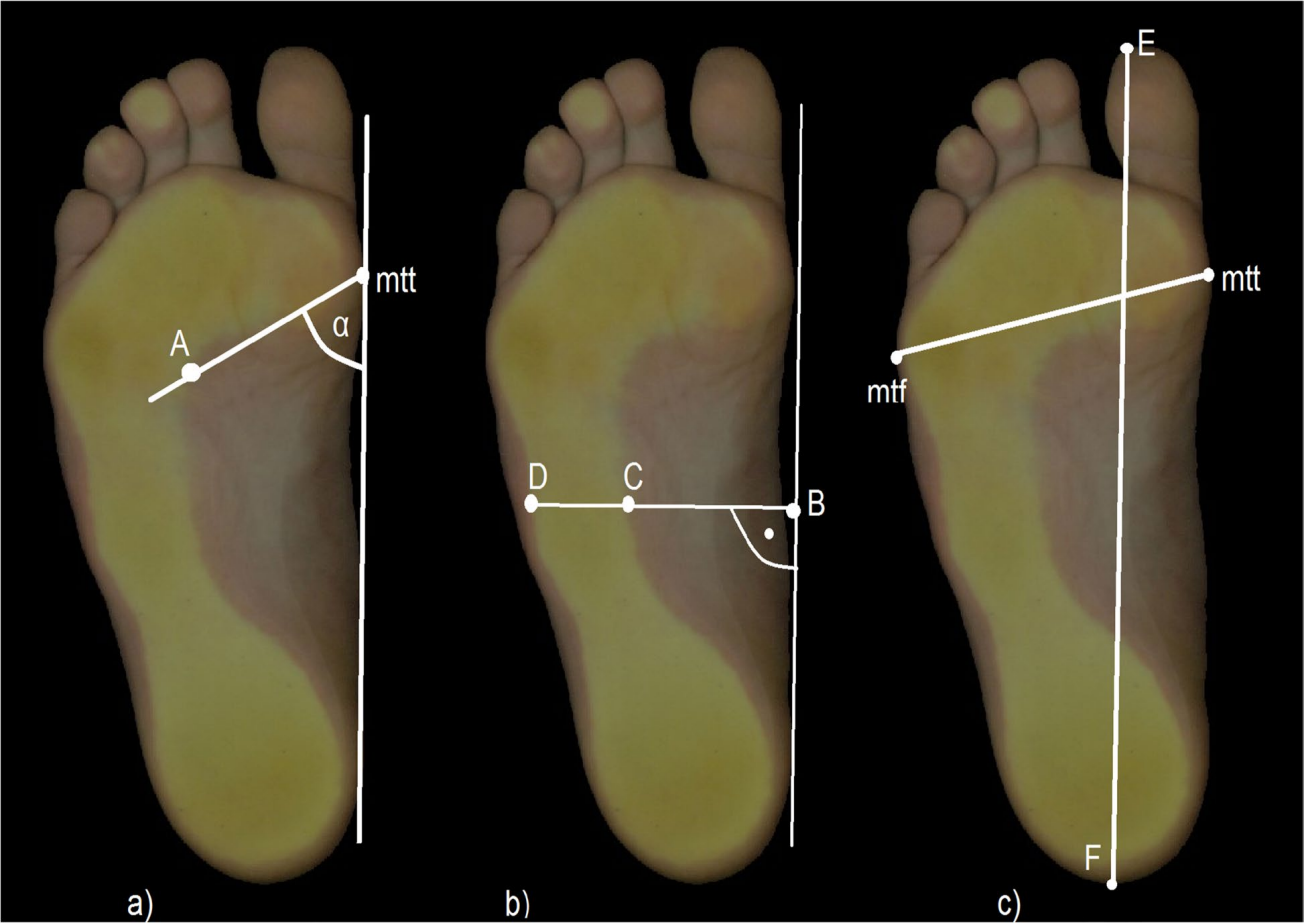
The method of determining the foot indicators on 2D footprint: **a**) Clarke’s angle, **b**) Sztriter-Godunov index, **c**) Wejsflog index. Source: Footprint obtained from 2D FootCAD PodoScan

To identify the transverse arch the following indicator was used:

- Wejsflog index (W index) – the ratio of the foot’s length (a line from the tip of longest toe [point E] to the extreme point of the heel [point F]) to its width (a line from point mtt to the most extreme point on the head of the fifth metatarsal bone [point mtf]) (Figure 2).

### Data analysis

Statistical analysis was performed using Dell Statistica 13.1 (TIBCO Software Inc., USA). The descriptive statistics are presented as mean values, standard deviation (SD), median (Med), ranges of variation (minimal [Min.] and maximal [Max.] values) and interquartile ranges (lower [Q_1_] and upper [Q_3_] quartiles). The categorical variables were calculated as frequencies (percentages). Normality of distribution was determined using the Kolmogorov–Smirnov test. In the data analysis Student’s *t*-test was used to assess the significance of the difference between the mean values of two independent groups; analysis of variance was also used. To ascertain the location of the difference, post hoc tests were used. The dependence between the selected variables was determined by the Spearman rank correlation test. For all tests, a significance threshold of *p* < 0.05 was used.

## Results

The programme entitled ‘Let’s get the kids moving’ included 337 boys and 318 girls. The mean age of the children was 8.7 years. The proportion of children at each CI level in our sample was as follows: underweight – 27.17% (*n* = 178); normal body weight – 51.14% (*n* = 335); overweight – 11.60% (*n* = 76); and obese – 10.07% (*n* = 66). The children’s characteristics are presented in Table 1 with respect to gender, age and CI category. The characteristics of the quality variables of the sample of 655 pupils are shown in Table 2.

**Table 1:** Descriptive characteristic of studied children

**Variables**	**Boys**	**Number, n (%)** **Girls**	**Total**
	337 (51.4)	318 (48.5)	655 (100)
**Age [years]**			
7	11 (3.2)	17 (5.3)	28 (4.3)
8	122 (36.2)	110 (34.6)	232 (35.4)
9	140 (41.5)	140 (44.0)	280 (42.7)
10	64 (19)	51 (16.0)	115 (17.6)
**Cole’s index category**			
Underweight	99 (29.4)	79 (24.8)	178 (27.17)
Normal body weight	179 (53.1)	156 (49.0)	335 (51.14)
Overweight	30 (8.9)	46 (14.5)	76 (11.6)
Obese	29 (8.6)	37 (11.6)	66 (10.07)

**Table 2:** Clinical characteristics for children

Variables	Boys Mean ± SD	Girls Mean ± SD	Total Mean ± SD	*p*-value
Age [years]	8.78 ± .83	8.71 ± .80	8.74 ± .79	> .05
Body weight [kg]	30.18 ± 6.90	29.79 ± 6.75	29.99 ± 6.83	> .05
Body height [cm]	135.05 ± 7.44	133.93 ± 6.80	134.51 ± 7.15	< .05
CI [%]	99.03 ± 15.18	101.03 ± 15.97	100.0 ± 15.6	> .05
CL ankle L foot [^o^]	46.73 ± 10.22	46.50 ± 9.05	46.60 ± 9.71	> .05
CL ankle R foot [^o^]	47.35 ± 9.31	47.75 ± 7.89	47.53 ± 8.71	> .05
K-y index L foot	.41 ± 0.21	.38 ± 0.20	.39 ± .21	< .05
K-y index R foot	.41 ± 0.20	.38 ± 0.18	.39 ± .19	< .05
W index L foot	2.77 ± 0.17	2.79 ± 0.15	2.78 ± .16	< .05
W index R foot	2.77 ± 0.16	2.79 ± 0.15	2.78 ± .16	> .05

*Abbreviations*: *SD* Standard deviation, *CI* Cole’s Index, *CL* Clark, *L* Left, *R* Right, *K-y* Sztriter-Godunov, *W* Wejsflog

### Longitudinal and transverse arches of the children’s feet

A tendency was noted for boys to have higher mean K-y index values than girls for both left (0.41 ± 0.21 vs 0.37 ± 0.20; *p* = 0.013) and right feet (0.41 ± 0.20 vs 0.38 ± 0.18; *p* = 0.005), while W index for left feet did not differ between genders (*p* = 0.045) (Table 3). There were no statistically significant differences between boys and girls or the mean values of CL angle for left and right feet, and of W index for left and right feet (*p* < 0.05).

**Table 3:** Gender differences in Clarke’s angle, Sztriter-Godunov index and Wejsflog index levels

	**Left foot**						**Right foot**					
**Gender**	**Mean ± SD**	**Min.-Max**	**Q**_**1**_	**Me**	**Q**_**3**_	***p-value***	**Mean ± SD**	**Min.-Max**	**Q**_**1**_	**Me**	**Q**_**3**_	***p-value***
**Clarke’s angle (CL angle)**												
Boys	46.73 ± 10.23	8–74	41	47	53	*p* = *0.407*	47.35 ± 9.31	4–76	42	48	53	*p* = *0.918*
Girls	46.50 ± 9.05	5–76	41	46	52		47.75 ± 7.89	23–73	42	47	52	
**Sztriter-Godunov index (K-y index)**												
Boys	0.41 ± 0.21	0–1	0.34	0.21	0.54	***p* = *0.013***	0.41 ± 0.20	0–1	0.35	0.45	0.52	***p* = *0.005***
Girls	0.37 ± 0.20	0–1	0.30	0.42	0.50		0.38 ± 0.18	0–0.84	0.31	0.41	0.49	
**Wejsflog index (W index)**												
Boys	2.79 ± 0.17	2.30–3.20	2.65	2.76	2.87	***p* = *0.045***	2.77 ± 0.16	2.31–3.20	2.67	2.78	2.88	*p* = *0.104*
Girls	2.79 ± 0.15	2.43–3.23	2.69	2.78	2.91		2.79 ± 0.15	2.41–3.19	2.69	2.79	2.89	

*Abbreviations*: *SD* Standard deviation, *Min.* Minimum value, *Max.* Maximum value, *Me* Median, *Q*_*1*_ Lower quartile, *Q*_*3*_ Upper quartile

^*^Significant *p*-values (< 0.05) are in bold

A statistically significant difference was found in mean CL angle for left feet in terms of CI score (*p* < 0.050) (Table 4). The detailed analysis established a significant difference in mean CL angle between the left feet of underweight and obese children and between those of normal weight and obese children. There were no significant differences in mean CL angle for right feet in terms of CI score (*p* < 0.050) (Table 4).

**Table 4:** Differences in Clarke’s angle, Sztriter-Godunov index and Wejflog index levels according to various Cole’s index categories

	**Left foot**						**Right foot**					
**Cole’s index (CI) category**	**Mean ± SD**	**Min.-Max**	**Q**_**1**_	**Me**	**Q**_**3**_	***p-value***	**Mean ± SD**	**Min.-Max**	**Q**_**1**_	**Me**	**Q**_**3**_	***p-value***
**Clarke’s angle (CL angle)**												
**Underweight**	48.52 ± 9.47	20–74	43	48	54	***p* < *0.050***	48.64 ± 9.08	23–72	43	49	54	0.158
**Normal body weight**	46.70 ± 9.38	5–76	41	47	53		47.16 ± 8.81	4–76	42	47	52	
**Overweight**	45.61 ± 8.88	25–64	40	46	51		48.22 ± 7.32	29–68	44	47	53	
**Obesity**	42.38 ± 11.07	15–62	36	42	49		45.76 ± 7.64	27–61	40	47	51	
**Sztriter-Godunov index (K-y index)**												
**Underweight**	0.35 ± 0.22	0–1	0.17	0.42	0.51	***0.002***	0.35 ± 0.21	0–1	0.26	0.42	0.49	***0.002***
**Normal body weight**	0.40 ± 0.20	0–1	0.33	0.44	0.51		0.40 ± 0.18	0–1	0.34	0.43	0.50	
**Overweight**	0.40 ± 0.18	0- 0.76	0.34	0.42	0.53		0.41 ± 0.17	0- 0.81	0.34	0.43	0.51	
**Obesity**	0.48 ± 0.18	0–1	0.38	0.48	0.55		0.46 ± 0.16	0–1	0.40	0.46	0.55	
**Wejsflog index (W index)**												
**Underweight**	2.82 ± 0.17	2.30–3.15	2.71	2.81	2.94	***p* < *0.050***	2.82 ± 0.16	2.31–3.2	2.73	2.81	2.94	***p* < *0.050***
**Normal body weight**	2.77 ± 0.16	2.32–3.23	2.67	2.77	2.88		2.78 ± 0.15	2.31–3.2	2.68	2.78	2.89	
**Overweight**	2.74 ± 0.14	2.48–3.1	2.63	2.72	2.84		2.74 ± 0.13	2.49–3.00	2.63	2.73	2.84	
**Obesity**	2.73 ± 0.16	2.37–3.1	2.59	2.77	2.85		2.73 ± 0 0.14	2.38–3.05	2.62	2.75	2.84	

*Abbreviations*: *SD* Standard deviation, *Min.* Minimum value, *Max.* Maximum value, *Me* Median, *Q1 *Lower quartile, *Q*_3_ Upper quartile

^*^Significant *p*-values (< 0.05) are in bold

There was a statistically significant difference in mean K-y index in terms of CI category of the left (*p* = 0.002) and right feet (*p* = 0.002) (Table 4). A more detailed analysis with the post hoc test revealed statistically significant differences in K-y index between the left feet of underweight and obese children and between those of normal weight and obese children. A statistically significant difference in K-y index value between the right feet of underweight and obese children, and between those of normal weight and obese children was observed. A very high positive correlation was found between the mean K-y index values for left and right feet (*p* < 0.05).

In underweight children, the mean W index value for left foot was significantly higher than among normal weigh, overweight and obese children. A similar statistically significant difference was found between the mean W index value for the right foot and CI category (*p* < 0.050). The underweight children had a significantly higher mean W index value for the right foot than their normal weight, overweight and obese peers (Table 4).

Statistical differences were revealed between the children’s age and the CL angle for the right foot (*p* < 0.050). No significant differences were found between the children’s age and the CL angle for the left foot, the K-y index for left and right feet or the W index for left and right feet (*p* > 0.05) (Table 5). The detailed analysis indicated significant differences in CL angle for the right foot between 7- and 8-year-olds, 07- and 10-year-olds and 9- and 10-year-olds (*p* = 0.039, *p* = 0.0007 and *p* = 0.045, respectively; data not presented).

**Table 5:** Clarke’s angle, Sztriter-Godunov index and Wejsflog index levels in various age groups

**Variables**	**7 year olds**		**8 year olds**		**9 year olds**		**10 year olds**		***p-value***
	**Mean ± SD**	**Min.-Max**	**Mean ± SD**	**Min.-Max**	**Mean ± SD**	**Min.-Max**	**Mean ± SD**	**Min.-Max**	
**CL angle – L foot**	43.59 ± 11.23	8–66	47.43 ± 10.22	9–76	46.03 ± 9.08	5–74	46.99 ± 9.66	15–69	*0.120*
**CL angle – R foot**	42.07 ± 11.04	9–60	47.43 ± 9.32	4–73	47.29 ± 7.99	19–76	49.55 ± 7.96	27–69	***p* < *0.050***
**K-y index – L foot**	0.39 ± 0.23	0- 0.94	0.40 ± 0.20	0–1	0.39 ± 0.20	0–1	.40 ± .21	0–1	*0.709*
**K-y index – R foot**	0.40 ± 0.23	0- 0.80	0.40 ± 0.18	0–1	0.39 ± 0.19	0–1	.40 ± .19	0–1	*0.919*
**W index – L foot**	2.81 ± 0.19	2.54–3.18	2.77 ± 0.16	2.32–3.23	2.78 ± 0.16	2.30–3.20	2.79 ± 0.16	2.35–3.15	*0.735*
**W index – R foot**	2.81 ± 0.19	2.45–3.20	2.78 ± 0.16	2.31–3.17	2.78 ± 0.15	2.31–3.20	2.79 ± 0.16	2.45–3.19	*0.722*

*Abbreviations*: *CL angle* Clarke’s angle, *K-y index* Sztriter-Godunov index, *W index* Wejsflog index, *SD* Standard deviation, *Min.* Minimum value, *Max.* Maximum value, *Me* Median, *Q*_*1*_ Lower quartile, *Q*_*3*_ Upper quartile, *L* Left, *R* Right

^*^Significant *p*-values (< 0.05) are in bold

In our study, we did not observe a statistically significant correlation between the children’s age and MLA or transverse foot arch, except for the CL angle for the right foot. A weak positive correlation (rho 0 = 0.109; *p* < 0.050) was found between the CL angle for the right foot and age.

## Discussion

In recent years, numerous studies from different countries have focussed on the paediatric foot in relation to weight category [9, 18–20]. In a systematic review of 34 studies describing the paediatric foot, Uden et al. [21] highlighted the fact that modern research lacks uniform guidelines for assessing foot posture. Many authors have taken up the issue of children’s excessive weight and foot arches [4, 9, 18, 12–24]. Nevertheless, few researchers include a group of underweight children in their analysis [8]. Mauch et al. [8] observed that underweight children had longer and more slender feet than children with healthy or excessive body weight. Moreover, Singh et al. reported that external tibial torsion can be associated with flexible paediatric flat foot [25].

Mauch et al. [8] observed 2887 students in Germany and found that flat feet were more common in overweight children. Jankowicz-Szymańska et al. [23] in a cross-sectional study conducted on 1377 preschool children, observed that Clarke’s angle correlated with weight category. These authors established that the MLA was highest in the normal weight children. Children between 3 and 7 years old who demonstrated excessive weight had a higher risk of developing flat feet than their peers at a healthy weight [23]. Maciałczyk-Paprocka et al. [22] reported similar correlations in a study among 2732 children. Obese children between the ages of 3 and 18 years were more predisposed to flat feet. It was suggested that childhood obesity is associated with structural foot and ankle deformities and activity-related foot pain. In addition to that, hallux valgus and high arches appeared to be heritable [26].

Corroborating our results, other authors [20, 27] have demonstrated that children with excessive weight more often have a flatter MLA than their normal body weight peers. The findings from a study performed among 550 Egyptian school children were that the obese pupils were 6.1 times more likely to have flat feet and 8.5 times more likely to experience foot pain [28]. The overweight and obese children with flat feet had higher levels of adipocyte cytokinesis than their peers with normal weight status [28]. Sadeghi-Demneh et al. [4] studied a group of 667 children aged 7 to 14 years and found that the obese children more often than their normal body weight peers experienced foot pain during physical activity. Woźniacka et al. [19] found a higher association between MLA and obese children among the girls.

Our finding that MLA was unchanging in early school-aged children is in line with other studies [5, 6, 24]. However, our research shows that CL angle for the right foot statistically significantly increases with the child’s age. This tendency for a stable foot arch structure was also confirmed by Hazzaa et al. [24] in a study on obese children ranging in age from 8 to 14 years.

Contrary to other researchers’ results [20, 24, 29], our study and one by Tong et al. [6] found no statistically significant differences between boys and girls in CL angle. In a cross-sectional study conducted on 2083 children from Taiwan between 7 and 12 years of age, Chang et al. [20] observed that the boys had higher tendency for flat feet than the girls. Hazzaa et al. [24], in a study among 150 obese children, observed that the boys had flat foot more often than the girls. Our results show that the boys had a higher mean K-y index than the girls. Puszczałowska-Lizis et al. [29] reported that girls had a higher mean CL angle than boys. Delgado-Abelan et al. [30], in their study of 1033 Spanish school-aged children, observed that foot dimensions were different between girls and boys at the age of 8 years. The contradictory results might be due to the use of different research tools and indexes for assessing flat foot in children.

Cobb et al. [31] reported that the value of Clarke’s angle had an impact on postural stability. They observed correlations between arch posture and mediolateral postural stability during a one-leg stance. In a study among 316 school children from Spain, Gijon-Nogueron et al. [32] found a high negative correlation between foot posture index and Clarke’s angle. Brzeziński et al. [33], in their large cohort study of 6992 children aged 8 to 12 years old, obtained similar results. They revealed that childhood obesity and overweight were connected with lower-limb postural defects. The feet of overweight and obese children had higher contact with the ground than those of their peers with normal body weight [34]. Children’s BMI negatively correlated with the centre of foot pressure [10]. Szczepanowska-Wołowiec et al. [10] found a statistically significant weak correlation between the CL angle of the right foot and the length and surface area of the centre of foot pressure. It is known that flat feet can cause functional instability of the foot, which is associated with the entire kinetic chain, balance and proprioception. This can lead to various dysfunctions in the knee joint, hip joint or spine as well as lower back pain [35, 36]. Abich et al. noted that children wearing closed shoes, with excess weight and a low level of physical activity were associated with flat foot development [37]. Because of the fact that early detection of abnormalities in the structure of the feet allows early rehabilitation or treatment incorporation, future disruption of the chain kinetic could be prevented. In addition to that, promoting physical activity may help reduce the risk of foot dysfunction in children [38].

Alsancak et al. [39] revealed that foot structures in Anatolian children aged 6 to 10 years differed depending on sex and age. Age, gender and body weight were associated with foot dimensions and pes planus diagnosis. The study revealed that, according to a classification made with the Staheli arch index, 63.3% had pes planus, 9.8% had pes cavus and 27.7% had a normal arch structure. There was a statistically significant correlation between BMI and increase in foot rotation in studied group. The association was stronger in girls than in boys [39]. Others factors might also affect the risk of a lower arch in children’s feet. Tong et al., in a study carried out on 111 healthy children with a mean age of 6.9 years, showed that children who walked in slippers at a younger age had a flatter MLA at a later stage of development than children who used sandals [6]. Cetin et al. [27], in research on 625 pupils between 6 and 13 years old, observed that the occurrence of flat feet in children was related to the place of residence and family history of flat foot.

One method for improving the arching of the feet is to practice short foot exercises [38]. A short foot exercise (SFE) is a special exercise that forms the basis for proprioceptive training of the foot. A regular SFE programme might improve this MLA and functional balance. The main purpose of SFE training is to practice an internal foot muscle contraction during different functional task with increasing difficulty [35]. The effect of muscular foot training while walking is to increase the amount of collagen in the tendons and ligaments of the feet, which increases the strength of the foot muscles.

### Future research

Researchers from our team have recognised the need for larger population studies to assess the morphology of children’s feet compared to those of their parents. In November 2020 we started the Population CohorT StUdy of Wroclaw CitizEns (PICTURE). It is planned for 2500 participants. In the PICTURE study, we conduct a re-examination two years after the first examination. Particular attention should be paid to the changing shape of arches in children with excessive body weight.

### Limitations

The limitations of the study include a lack of data on musculoskeletal symptoms among overweight and obese children with lower foot arches. Further limitations were the limited number of participants in the study and the fact that confounding variables were not controlled for. The confoundinger variables included: plantar fascia thickness, foot arch height and, plantar pressure in the big toe and heel, and eversion of ankle strength. A lack of consensus regarding what constitutes a typically developing paediatric foot limits the ability of clinicians to appropriately diagnose and manage their patients. It is unclear what importance, if any, should be attached to the static posture of the developing foot, considering the noticeable lack of functional and clinical data. The use of footprint indices in those with higher BMI readings values results in a larger contact area due to the increased adipose tissue around the arch, which when measured as a 2D construct skews data to suggest a flatter foot. When the foot is measured as a 3D construct (e.g. using the Foot Posture Index—6), no difference in foot posture is observed between overweight/obese and normal weight children. Given the increased levels of adipose tissue in children irrespective of foot posture, footprint indices of any type do not offer an adequate means to establish anything other than the shape of the arch. Therefore, the Foot Posture Index (FPI-6) is considered the measure of choice. However, the FPI-6 does not allow for a complete assessment of the foot in all planes and has limited use in the diagnosis of certain acquired and congenital diseases of the feet. There is still currently no single reliable test to assess whether to initiate treatment and to indicate the optimal type of treatment depending on the measurements.

## Conclusions

Examination of feet in the 2D construct enables the initial detection of abnormalities in the shape of the arch. However, the differences in the shape of the arch between children with normal and overweight body mass require further research. Children may experience greater limitations participating in a wide range of physical activities, which may lead to social isolation. When conducting screening examinations to assess children’s body posture, researchers ought to look at each child holistically and consider the entire body posture parameters and weight status, not just measurements of the feet. An examination of every child suspected of having abnormalities in the foot arches should additionally include functional tests to check the muscular capacity of the child’s foot.

## Data Availability

The datasets used and/or analysed during the current study are available from the corresponding author on reasonable request.

## Abbreviations

CCD: Charge Coupled Device
CL: Clarke’s angle
CI: Cole’s Index
K-y index: Sztriter–Godunov index
L: Left
Max.: Maximum value
MDD: Medical Device Directive
Med: Median; Min.: minimal value
MLA: Medial longitudinal arching
mtf: Metatarsale fibulare
mtt: Metatarsale tibiale
PICTURE: Population Cohort Study of Wroclaw Citizens
Q_1_: Lower quartile
Q_3_: Upper quartile R: right
SD: Standard deviation
SFE: Short foot exercise
STROBE: Strengthening Reporting of Observational Studies in Epidemiology
W index: Wejsflog index
